# Chronic Pancreatitis in Bristol

**Published:** 1983-07

**Authors:** Sulieman S. Fedail, P. R. Salmon, R. F. Harvey, A. E. Read

**Affiliations:** University Department of Medicine Bristol Royal Infirmary; University Department of Medicine Bristol Royal Infirmary; University Department of Medicine Bristol Royal Infirmary; University Department of Medicine Bristol Royal Infirmary


### Chronic Pancreatitis in Bristol

**Sulieman S. Fedail,* M.D.**
**P. R. Salmon, F.R.C.P.**
**R. F. Harvey, M.D., F.R.C.P.**
**A. E. Read, M.D., F.R.C.P.**
University Department of Medicine Bristol Royal Infirmary

### INTRODUCTION

Chronic pancreatitis is being increasingly diagnosed in the United Kingdom, and there is an impression that its incidence is rising. Studies from London[1] and Manchester[2] support this impression. However, these previous studies were retrospective. A prospective study was therefore carried out over a period of 30 months (January 1976–June 1978) of all the cases of chronic pancreatitis admitted to the University Department of Medicine at the Bristol Royal Infirmary.

The primary objective of the study was to investigate the aetiology and clinical manifestations of chronic pancreatitis.

### MATERIALS AND METHODS

A total of 30 patients were seen, 23 males and 7 females. The mean age was 44 years (range 20–70). Only patients living in the Bristol health district (total population 330,000) were included. All the patients were interviewed by one of us (S S F) using a special questionnaire. The diagnosis of chronic pancreatitis was established by two or more of the following criteria: abnormal endoscopic retrograde pancreatography (ERCP),[3] abnormal secretin test,[4] and abnormal Lundh test meal.[5]

Alcohol intake was regarded as heavy and thus likely to be of aetiological significance if daily consumption was greater than one bottle of wine, half a bottle of spirits (eg. whisky) or eight pints of beer.[1] Full blood count, plasma viscosity, liver function tests, serum amylase and calcium and three day faecal fat excretion were measured in all patients.

### RESULTS

During the period of this study, 30 patients were seen who fulfilled the criteria for the diagnosis of chronic pancreatitis (Table 1). The likely aetiological factors are shown in Table 2. Alcohol seemed to be more likely as an aetiological factor in young patients (high intake in 10 of 13 patients under 40 years of age, compared with 6 of 17 aged over 40).

**Table 1**

**Investigations used to establish the diagnosis of chronic pancreatitis**

| *Investigation* | *No. of patients investigated* | *Patients with abnormalities No.* | *%* |
|---|---|---|---|
| Secretin test | 28 | 25 | 89 |
| Lundh test | 29 | 22 | 76 |
| ERCP | 30 | 27 | 90 |

**Table 2**

**Aetiological factors in chronic pancreatitis**

| *Cause* | *No.* | *%* |
|---|---|---|
| High alcohol intake | 16 | 53 |
| Idiopathic | 10 | 34 |
| Isolated ventral pancreas | 2 | 7 |
| Gall stones | 1 | 3 |
| Primary hyperparathyroidism | 1 | 3 |

Physical signs on examination of these patients were very few. Jaundice was present in two patients with concomitant alcoholic cirrhosis. Tenderness in the epigastrum was elicited in four patients, and another four patients had hepatomegaly (two diabetic and two with cirrhosis).

All the haematological investigations, haemoglobin, white blood count and viscosity were normal. The liver function tests were abnormal in the two patients with cirrhosis of the liver. The serum calcium was elevated in one patient who later was shown to have hyperparathyroidism. Streatorrhoea (faecal fat more than 15 mmols per day) was found in eight patients. Four patients had calcification in the pancreas.

* Present address: Department of Medicine, Faculty of Medicine, University of Khartoum, Khartoum, Sudan.

### DISCUSSION

The mean age at onset of chronic pancreatitis in this series was 44 years, which is higher than that reported from Manchester (31.7 years),[2] but similar to the mean age reported from London (46.7 years).[1] It is also higher than the mean age reported from France (38.4 years).[6] In accordance with the other studies, there were more males than females.

Alcohol intake was difficult to assess exactly, as patients tend to refuse to disclose the full extent of their alcohol consumption. 53% were regarded as having a heavy alcohol intake, and alcohol was considered as the main aetiological agent of their chronic pancreatitis. This may be an underestimate of the extent of the problem. However, it is similar to the figure of 55% reported from Manchester[2] and the 42% reported from London.[1] Still the incidence is far less than the incidences reported from France[6] and the United States.[7] This is higher than the incidences previously reported from the United Kingdom.[8,9] This rising proportion of alcoholic pancreatitis parallels the rise in alcohol consumption in the last few years.[10] The other important finding is that the majority of cases of alcoholic pancreatitis were in the young age group. 10 out of the 13 in the age group 20 – 40 years drink heavily, and alcohol was regarded as the main aetiological agent of their chronic pancreatitis.

Two cases had congenital ventral pancreas which is now known to be associated with chronic pancreatitis.[11] Gallstones were responsible for one case only, this is in line with the general consensus that gallstones very rarely cause chronic pancreatitis.[12] One patient had primary hyperparathyroidism, she had a high serum calcium and at surgery a parathyroid adenoma was found. Hyperparathyroidism is a recognised cause of chronic pancreatitis and this case underlines the importance of measuring serum calcium in all cases of chronic pancreatitis as it is a potentially curable cause.

Analysis of the symptoms (Table 3), showed that abdominal pain was the commonest symptom, two cases only had painless pancreatitis. This is similar to the incidence of 5–10% reported in the literature.[13]

**Table 3**

**Commonest symptoms at presentation**

| *Symptom* | *Patients* *No.* | *%* |
|---|---|---|
| Abdominal pain | 28 | 93 |
| Belching | 22 | 73 |
| Nausea & vomiting | 18 | 60 |
| Weight loss | 17 | 57 |
| Diarrhoea | 5 | 17 |

When the features of abdominal pain in this series (Table 4) were compared with the features of abdominal pain in France,[6] some differences were found. The incidence of recurrent abdominal pain was higher in the French series, 82%, whereas it was 71% in our series. Radiation of the pain to the back occurred in 57% of our cases whereas no mention of radiation of pain to the back was found in the French series. However, a more recent study from France[14] reported radiation of pain to the back in 56% of patients. Relief of pain by bending forward occurred in only 14% of our cases, whereas it occurred in 62% of patients in the French series. These differences might be partly due to the higher incidence of alcoholic pancreatitis in France, resulting in a severe disease. Recurrent pain comes on with recurrent alcohol consumption. The character of the pain was described as dull and steady in 39% of cases, sharp in 39% of cases and only 3% described it as colicky which emphasises the rarity of colicky pain in chronic pancreatitis.[8] The duration of the pain was more than 12 hours in 83% of the cases, which may help to differentiate between it and biliary colic.

**Table 4**

**Features of abdominal pain in patients with chronic pancreatitis**

| | *No. of cases* | *%* |
|---|---|---|
| Abdominal pain (present in 28/30 cases): | | |
| Constant | 8 | 29 |
| Recurrent | 20 | 71 |
| Site of pain: | | |
| (a) Diffuse epigastrium | 16 | 57 |
| (b) Central | 9 | 32 |
| (c) Right hypochondrium | 2 | 7 |
| (d) Left hypochondrium | 1 | 3 |
| Character of pain: | | |
| (a) Steady and dull | 11 | 39 |
| (b) Sharp | 11 | 39 |
| (c) Burning | 4 | 14 |
| (d) Colicky | 2 | 7 |
| Radiation to the back | 16 | 57 |
| Relief of pain by bending forward | 4 | 14 |
| Duration of pain more than 12 hours | 25 | 83 |

The next common symptom was belching, which occurred in 73% of cases. This was rather surprising, though Howat[8] back in 1963 wrote 'painful eructation is often troublesome when the tail of the pancreas was involved'. However, none of our patients had isolated involvement of the tail of the pancreas.

Weight loss occurred in 57% of cases. Abdominal pain seems to be the major cause for weight loss, as it deters the patient from eating. Pain occurred in all patients who had weight loss. The other causes of weight loss are not common. Diabetes mellitus occurred in two cases and both had weight loss. Streatorrhoea was demonstrated in eight cases and of these only three had weight loss.

Diarrhoea occurred in 17% of cases, similar to the incidence reported from Ireland.[15] All had increased faecal fat.

Our study confirmed the fact that physical signs are very few or absent in chronic pancreatitis, the physical findings in our cases were those of concomitant disease.

This study seemed to confirm the impression that the incidence of chronic pancreatitis is rising in the United Kingdom and that alcohol is the major aetiological agent.

### ACKNOWLEDGEMENT

We are very grateful to the various consultants who referred their cases to us and to the members of the Department of Medicine who helped us to study these cases.

### REFERENCES

1. JAMES, O, AGNEW, J. E., BOUCHIER, I. A. D. (1974) Chronic pancreatitis in England: a changing picture? *BMJ* **2**, 34–38.
2. READ, G., BRAGANZA J. M., HOWAT, H. T., (1976) Pancreatitis – a retrospective study. *Gut,* **17**, 945–952.
3. KASUGAI, T., KUNO, N., KIZU, M. (1974) Manometric endoscopic retrograde pancreatocholangiography, technique, significance and evaluation. *Am.J.Dig.Dis.* **19**, 485–502.
4. DREILING, D. A., JANOWITZ, H. D. (1962) The measurement of pancreatic secretory function. In: REUCK, A. V. S., CAMERON, M. P., Eds Ciba Foundation symposium on the exocrine pancreas. London: Churchill, pp. 225–252.
5. MOTTALEB, A., KAPP, F., NOGUREA, E. C. A., KELLOCK, T. D., WIGGINS, H. S., WALLER, S. (1973) The Lundh test meal in the diagnosis of pancreatic diseases: A review of five years' experience. *Gut,* **14**, 835–841.
6. SARLES, H., SARLES, J., CALMATTEE R. et al. (1965) Observations on 205 confirmed cases of acute pancreatitis, recurring pancreatitis, and chronic pancreatitis. *Gut,* **6**, 545–559.
7. OWENS, J. L., HOWARD, J. M. (1958) *Annals of Surgery,* **147**, 326.
8. HOWAT, H. T. (1963) Chronic pancreatitis: *The Practitioner* **191**, 42–49.
9. HOWAT, H. T. (1968) Chronic pancreatitis: medical aspects. *Postgrad Med.J.,* **44**, 733–736.
10. Report of the departmental committee on liquor licensing 1972; London, HMSO.
11. COTTON, P. B. (1980) Congenital anomaly of pancreas divisum can cause obstructive pain and pancreatitis. *Gut,* **21**, 105–114.
12. SARLES, H., GEROLAMI-SANTANDREA, A. (1972) Chronic pancreatitis. *Clin Gastroenterol* **1**, 167–193.
13. FITZGERALD, O. (1972) Painless pancreatitis and other painless pancreatic disorders. *Clin. Gastroenterol* **1**, 195–218.
14. SARLES, H., SAHEL, J., STAUB, J. L., BOURRY, J., LAUGIER, R. (1979) Chronic pancreatitis. In: HOWAT, H. T. and SARLES, H., Eds: The Exocrine Pancreas. London, W. B. Saunders; 402–435.
15. FITZGERALD, O., FITZGERALD, P., FENELLY, J., MACMULLIN, J. P., BOLAND, S. (1963) A clinical study of chronic pancreatitis. *Gut,* **4**, 193–216.

